# Dapagliflozin lowers blood pressure via regulating the sympathetic neural activity in the paraventricular nucleus of hypothalamus in normal mice

**DOI:** 10.3389/fendo.2026.1689167

**Published:** 2026-01-23

**Authors:** Meiyuan Dong, Zhimin Xu, Ligang Zhou, Song Wen

**Affiliations:** 1 Department of Endocrinology, Shanghai Pudong Hospital, Fudan University, Shanghai, China; 2 Shanghai Key Laboratory of Vascular Lesions Regulation and Remodeling, Shanghai Pudong Hospital, Fudan University, Shanghai, China; 3 Fudan Zhangjiang Institute, Fudan University, Shanghai, China

**Keywords:** blood pressure, central nervous system, paraventricular nucleus, sodium-glucose cotransporter-2 inhibitors, sympathetic nervous system

## Abstract

**Background and aims:**

The risk of heart failure (HF) is significantly higher in patients with type 2 diabetes mellitus (T2DM). Recent studies have shown that an imbalance in the sympathetic nervous system (SNS) is a target for sodium-glucose cotransporter-2 inhibitors (SGLT-2i), which have been proven to reduce the risk of adverse HF outcomes in several clinical trials. However, the specific central mechanisms involved remain unclear. Therefore, our research investigates how dapagliflozin (DAPA) regulates blood pressure through SNS and explores the central neural and endocrinological pathways by which DAPA, an SGLT-2i, influences SNS activity and its impact on the cardiovascular system in the treatment of HF in T2DM.

**Material and methods:**

Twenty-one male C57BL/6 mice aged 8–10 weeks were randomly divided into three groups (n = 7 each): control (CTRL), DAPA, and DAPA-Cyanine 3 (DAPA-Cy3). Blood pressure (BP), heart rate (HR), and blood glucose (BG) were measured after a single dose of treatment. DAPA-Cy3 was designed to assess its ability to cross the blood-brain barrier (BBB). Additionally, histological co-localization analyses of immunoreactivity for c-Fos, neuronal nitric oxide synthase (nNOS), corticotropin-releasing hormone (CRH), and vasopressin (VP) across groups were performed to explore how SGLT-2 inhibitors regulate the central sympathetic nervous system (SNS).

**Results:**

1) DAPA significantly reduced both systolic blood pressure (SBP) and diastolic blood pressure (DBP) in mice, without notably affecting the heart rate (HR); 2) SGLT-2 was found to be extensively expressed in the central nervous system (CNS), especially in the hypothalamus and brainstem; 3) DAPA-Cy3 was able to cross the blood-brain barrier (BBB) and disperse throughout the brain; and 4) DAPA clearly influenced the activity of specific neurons expressing nNOS, CRH, and VP in the hypothalamus.

**Conclusion:**

DAPA, as an SGLT-2 inhibitor, crosses the BBB and binds to the innate SGLT-2 in the hypothalamus and brainstem of mice. This significantly influences the tone of the SNS through indirect regulation by modulating nNOS, CRH, and VP, which are believed to be the upstream regulatory points of SGLT-2 in interacting with the SNS.

## 1 Introduction

Poor glycemic control in Type 2 diabetes mellitus (T2DM) patients eventually leads to cardiovascular complications such as HF, stroke, myocardial infarction, and various adverse outcomes (1, 2). The key pathophysiological mechanism involves: 1. The autonomic nervous system (ANS), especially the sympathetic nervous system (SNS), which plays a crucial role in the development of hypertension (HT) and HF, shows overactivity in T2DM, obesity, and metabolic syndrome (Met) (3); 2. Long-term studies in animals and humans suggest that increased hypothalamic SNS activity may be involved in the development of HT and CVD (4); 3. Overactivity of the renin-angiotensin-aldosterone system (RAAS) has also been shown to be linked to the effects of the central SNS, impaired regulation of cardiovascular function, and activity, which worsens the overactivity of both the SNS and RAAS (5).

**Figure 1:**
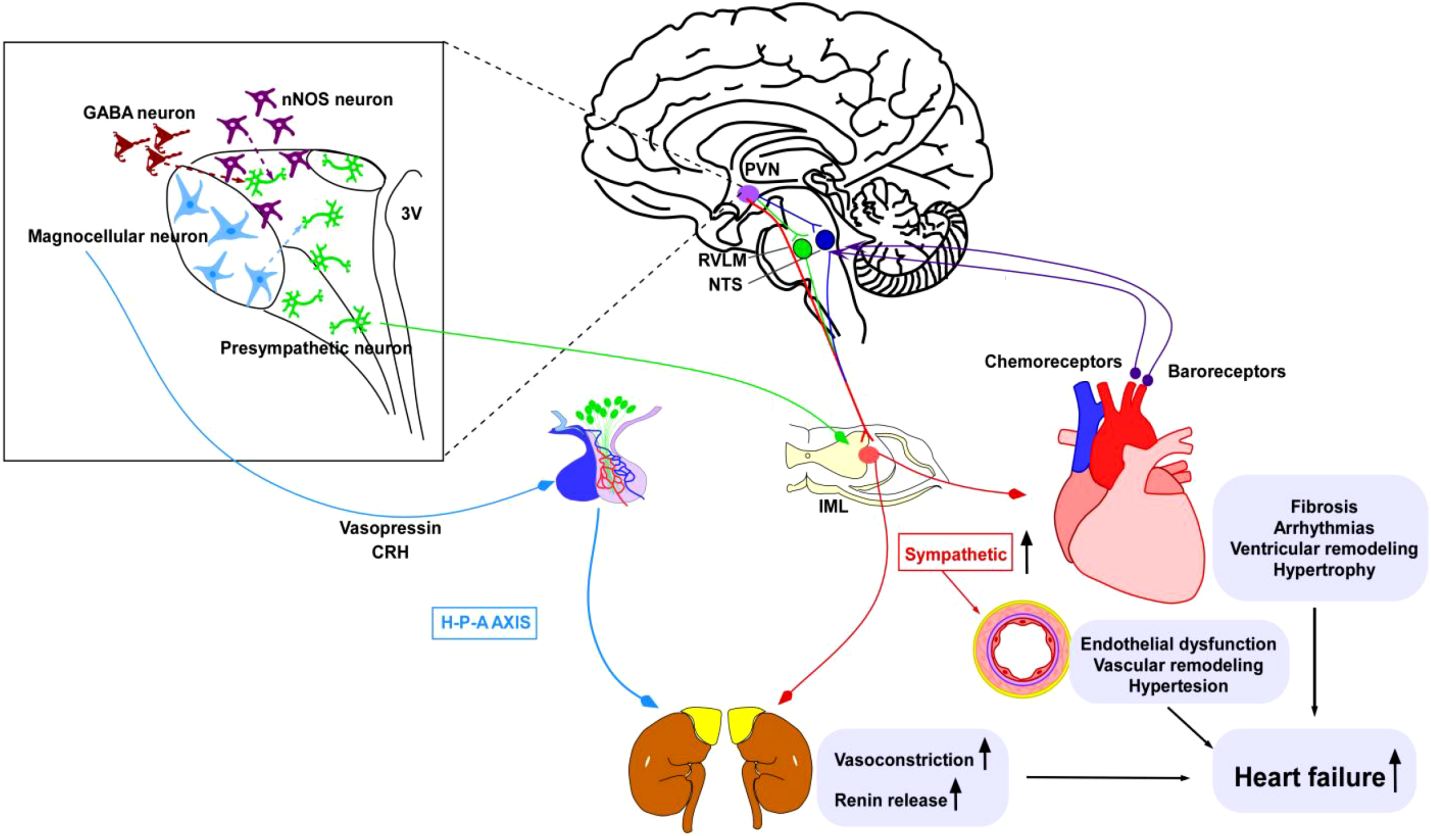
The current understanding of the relationship between HF and the regulation of SNS and CNS nuclei responsible for cardiovascular control. This interconnection includes pre-sympathetic neurons, their upstream GABA and nNOS neurons, as well as magnocellular neurons that regulate the VPN and CRH. Their converged targets are the kidney and adrenal gland, which respond by eliciting vasoconstriction and renin release to increase blood pressure. Another downstream target organ is the heart, which translates chemoreception and baroreception information to the brainstem nuclei rostroventrolateral medulla (RVLM) and nucleus of the solitary tract (NTS). The heart receives sympathetic tone from the intermediolateral nucleus (IML) of the spinal cord, where pre-sympathetic neurons reside. Enhanced sympathetic tone can lead to endothelial dysfunction, vascular and myocardial remodeling, and hypertension — the pathophysiological basis for heart failure.

The paraventricular nucleus of the hypothalamus (PVN) consists of pre-sympathetic neurons projecting to the spinal cord and those controlling the hypothalamic-pituitary-adrenal (HPA)) axis. Corticotropin-releasing hormone (CRH) neurons in the PVN are central players in the stress response, integrating external and visceral stress-related information. Through output from the PVN, pre-sympathetic neurons control the activity of the autonomic nervous system (ANS), and the release of CRH diffusively influences the endocrine system (6). In addition, nNOS neurons are expressed throughout the central and peripheral nervous systems, acting on different parts of the CNS, including the PVN and NTS (7). It also exhibits a sympathoinhibitory effect on a range of diseases, including heart failure (HF), hypertension (HT), and type 2 diabetes mellitus (T2DM) (8). On the other hand, vasopressin (VP) is one of the key neuropeptides involved in regulating cardiovascular function and blood pressure (BP). It is primarily synthesized in the supraoptic nucleus (SON) and PVN within the hypothalamus, functioning as both a hormone and a neurotransmitter in the CNS (9). Recent evidence suggests that the VP released from the hypothalamic PVN is associated with sympathetic nervous system (SNS) overactivity (10). Experiments have demonstrated that VP can increase BP and heart rate (HR). Administering VP within the PVN in normotensive rats significantly elevates BP and induces an increase in SNS activity (11, 12) (**Figure 1**).

It is well known that SGLT-2 inhibitor (SGLT-2i) primarily reduces renal glucose reabsorption by inhibiting SGLT-2 in the proximal tubules, thereby exerting significant glucose-lowering effects (13, 14). Notable therapeutic benefits for hypertension and heart failure have been observed in Cardiovascular Outcome Trials (CVOTs) involving patients on long-term SGLT-2i treatment, which are separate from its antidiabetic effects (15, 16). Furthermore, emerging research has emphasized the potential inhibitory effect of SGLT-2i on SNS activity, underlying the mechanism of diabetic cardiovascular therapy (17, 18). Interestingly, recent studies have also shown that in a non-diabetic prehypertensive rat model, SGLT-2i Dapagliflozin (DAPA) reduced resting blood pressure and SNS activity (19). Therefore, DAPA, as an SGLT-2 inhibitor, could be a promising candidate for treating cardiovascular disease. However, the precise mechanism by which it lowers SNS activity remains unclear. Previous studies have mainly focused on its direct effects on peripheral sympathetic nerve excitability, with little attention to central mechanisms. Thus, we aim to explore the relationship between the pharmacological properties of DAPA and its central effects by investigating its ability to permeate the blood-brain barrier (BBB), its binding regions and patterns within the brain associated with autonomic nervous system (ANS) control, and via c-Fos analyses, elucidate the mechanism of neuron activation related to DAPA’s action. Additionally, we examined how pre-sympathetic neurons such as nNOS, CRH, and VP within the hypothalamus upstream of the PVN are modulated by DAPA and explored the potential brain-renal axis, including access to the ANS and RAAS, that may contribute to HF treatment.

## 2 Materials and methods

### 2.1 Animals

Adult male C57BL/6 mice, 8–10 weeks old and weighing approximately 20 g, were used in this study. The mice were purchased from Shengchang Biotechnology Co., Ltd. Before the experiment, the animals were acclimated for one week at the animal experimental center of Shanghai Pudong Hospital. They were housed in groups under constant temperature (20 ± 2 °C), humidity (40 ± 10%), and a 12/12-hour light/dark cycle. The animals had free access to purified water and food throughout the study. The protocol was approved by the Ethics Committee for Animal Experiments and adhered to relevant laws and regulations for laboratory animals in China. The decision to utilize only male mice in this initial, proof-of-concept study was primarily made to minimize experimental variance and to establish a clear foundational understanding of DAPA’s central mechanisms within a uniform physiological context.

### 2.2 Drugs and chemicals

Dapagliflozin (AstraZeneca) was obtained from Shanghai Pudong Hospital. Using dapagliflozin (B21884), dapagliflozin-Cy3 (DAPA-Cy3) was synthesized by Shanxi Carebio Biosciences Company. DMSO (1.52%) was used to dissolve each of the tested agents (DAPA and DAPA-Cy3). Dapagliflozin and DAPA-Cy3 were administered orally to the mice.

## 3 Methods

### 3.1 BG measurement

Prior to the experiment, mice were fasted for 12 hours. Blood glucose (BG) levels in the tail vein were measured at 0 hours. Immediately after the measurement, the experimental (DAPA) and control (CTRL) groups received 1 mg/kg DAPA and an equal volume of saline via intragastric administration. BG levels were measured using a Roche glucometer (calibrated before use) at 0.25, 0.5, 0.75, 1, and 2 hours after administration. The related methodology can be found in (20).

### 3.2 Measurement of BP and HR

Systolic blood pressure (SBP), diastolic blood pressure (DBP), and HR were measured at 0 hours. Mice in both experimental and control groups received 1 mg/kg DAPA or an equivalent volume of saline via gavage. Two hours later, BP and HR were measured and recorded. Blood pressure (BP) and heart rate (HR) were measured using a CODA-Monitor (21).

The imaging of localization of DAPA-Cy3 in kidney and brainstem

Mice were intragastrically given 1 mg/kg dapagliflozin-Cy3. Two hours later, they were anesthetized with isoflurane inhalation, and their kidneys and brain tissues were collected. The tissues were rinsed with PBS to remove surface blood, and their surface moisture was wiped off with lint-free paper before imaging. Then, frozen sections (25 μm) of the mouse brains were prepared, and the distribution of dapagliflozin-Cy3 was observed under a microscope. The rationale and utility of this equipment are explained in (22).

### 3.3 Western blotting

Total protein was extracted with RIPA buffer. A was used to resolve the sample using 7.5-12% SDS-PAGE. Tris-buffered saline with Tween 20 and 3% bovine serum albumin (BSA) were used for membrane blocking at 25 ± 2 °C for 1 h, followed by incubation at 4 °C overnight. Finally, the samples were visualized by chemiluminescence using a ChemiDoc MP imager. The results were expressed as arbitrary units after normalization to β-actin protein expression. The levels of c-Fos, CRH, and VP were measured using this method. The methods have been described in detail in (23).

### 3.4 Immunohistochemistry and immunofluorescence

After inducing deep anesthesia, the mice underwent transcardial perfusion with 20 ml of 0.9% normal saline, followed by 20 ml of 4% paraformaldehyde. The mice’s brains were carefully dissected and then preserved in 4% paraformaldehyde at 4 °C overnight for fixation before being transferred to a 30% sucrose solution. The brain tissue was subsequently sliced into 30 μm-thick sections and collected in 0.01 M PBS. On the first day, the frozen sections were rinsed three times in PBS for 5 minutes each. Next, the sections were permeabilized with 0.3% Triton X-100 solution, then rinsed three more times with PBS for 5 minutes each. Sections were blocked with 5% goat serum at room temperature for 1 hour. They were then incubated overnight at 4 °C on a shaker with solutions of mouse anti-c-Fos antibody (1:1000, Abcam), rabbit anti-CRH antibody (1:500, Abcam), and mouse anti-SGLT-2 antibody (1:200, Abcam) diluted in PBS. On the second day, the tissue sections were rinsed three times in PBS for 5 minutes each. Then, they were incubated for 1 hour at room temperature with goat anti-mouse H&L (Alexa Fluor^®^ 594, 1:1000, Abcam) or goat anti-rabbit H&L (Alexa Fluor^®^ 488, 1:1000, Abcam), diluted in PBS. After incubation, the sections were rinsed three times with PBS for 5 minutes each. Once rinsed, the brain slices were mounted onto glass slides and sealed with an anti-fluorescence quenching agent. A similar method can be found in (24).

### 3.5 Injection of retrograde tracers

Mice were anesthetized with Zoletil-50 at a dose of 0.1 ml/10 g, and 4% fluorogold (FG) was injected into their kidneys. After 14 days, mice received dapagliflozin (1 mg/kg) via oral gavage. Two hours later, perfusion and tissue sampling were performed. The rationale for this technique can be found in (25).

## 4 Statistical analysis

All experimental data are expressed as mean ± SEM and were analyzed statistically using GraphPad Prism 9 and SPSS 26.0. The t-test was employed for comparisons between two groups, while one-way ANOVA or two-way ANOVA with the Bonferroni *post-hoc* test was used for multiple group comparisons. Image processing was performed using ImageJ software. However, Mead’s Journal Pre-proof ‘Resource Equation’ was used to ensure that sample sizes are sufficient to demonstrate a statistically significant difference (26).

All histological image analysis and Western Blot band quantification were performed under single-blind conditions, where the operators were unaware of the experimental group assignments to minimize analytical bias.

## 5 Results

### 5.1 Blood glucose, blood pressure and heart rate

**Figure 2:**
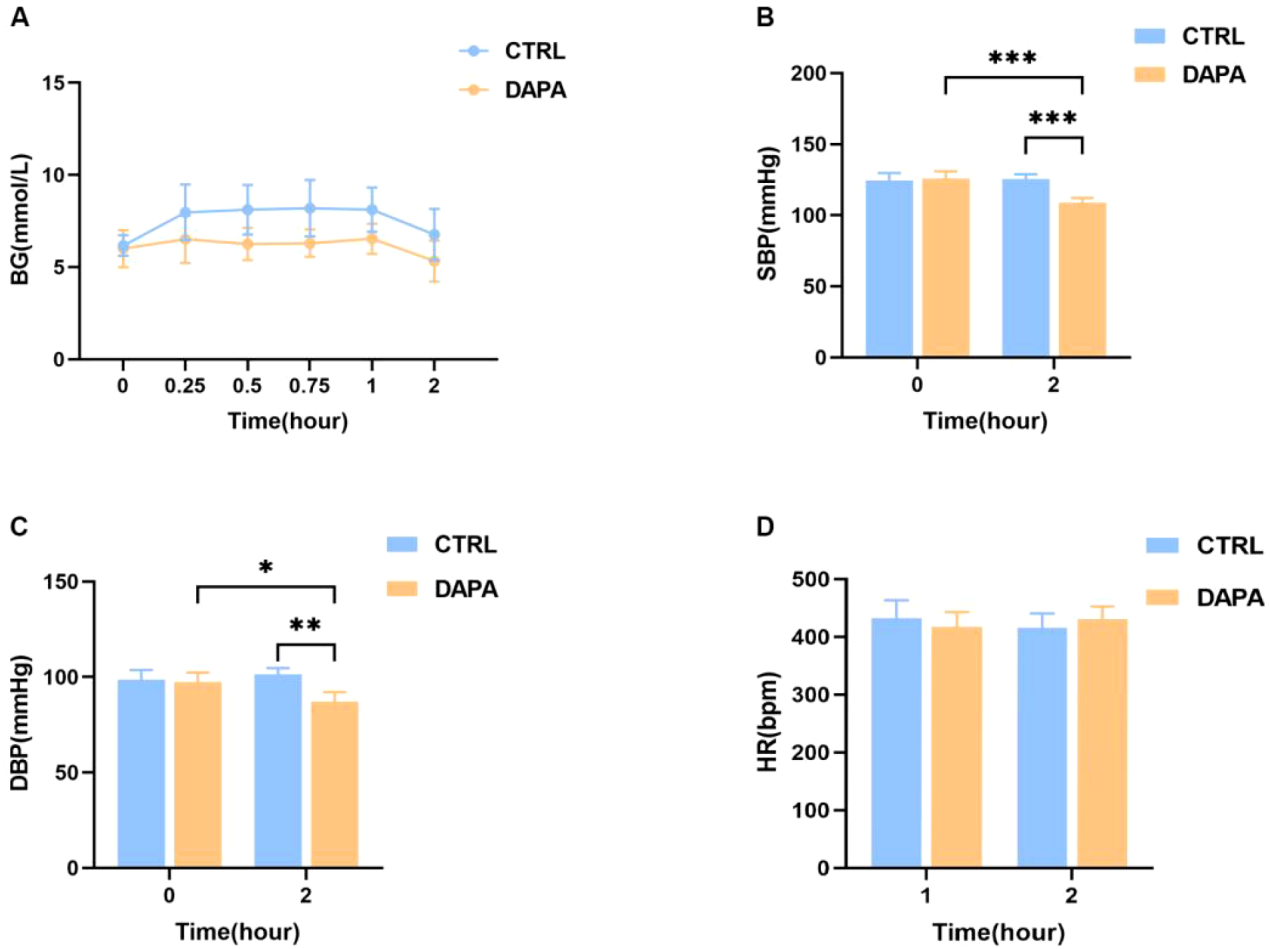
The comparison of BG, BP and HR after administration of Dapagliflozin or saline. **(A)** BG testing performed 2 hours after administration showed that both the DAPA and control groups had BG levels within the normal range. Importantly, there was no statistically significant difference in overall BG levels between the two groups (P > 0.05, n=7). **(B)** After 2 hours, the SBP in the DAPA group was significantly lower than in the control group (n=7). Additionally, the SBP in the DAPA group at 2 hours post-administration was significantly reduced compared to pre-administration levels. **(C)** After 2 hours, the DBP in the DAPA group was significantly lower than in the control group. Moreover, the DBP in the DAPA group at 2 hours post-administration was significantly decreased compared to pre-administration levels. **(D)** No significant differences in HR were observed between the DAPA and control groups at both 0 hours and 2 hours. Data are shown as mean ± SEM. Note: *P < 0.05, **P < 0.01, ***P < 0.001 versus indicated groups.

BG, HR, SBP, and DBP measurements are shown in **Figure 2**. There was no statistically significant difference in BG concentrations between the DAPA and control groups (Mean ± SEM: DAPA vs. Ctrl: 0 min: 6.000 ± 0.37859 mmol/L vs. 6.171 ± 21125 mmol/L; 0.25 h: 6.5143 ± 0.48375 mmol/L vs. 7.9714 ± 0.57101 mmol/L; 0.5 h: 6.2571 ± 0.33228 mmol/L vs. 8.1143 ± 0.50729 mmol/L; 0.75 h: 6.3000 ± 0.28200 mmol/L vs. 8.2000 ± 0.57982 mmol/L; 1 h: 6.5429 ± 0.30927 mmol/L vs. 8.1143 ± 0.45378 mmol/L; 2 h: 5.3286 ± 0.41731 mmol/L vs. 6.7714 ± 0.52541 mmol/L; P>0.05); although the BG of mice in the DAPA group decreased (**Figure 2A**). The results in **Figures 2B, C** show that the SBP of mice given dapagliflozin significantly decreased compared to the control group after 2 hours (SBP: Mean ± SEM: DAPA vs. Ctrl: 0 h: 125.7800 ± 1.94062 mmHg vs. 124.5568 ± 1.96362 mmHg, P>0.05; 2 h: 108.8923 ± 1.23878 mmHg vs. 125.6131 ± 1.26808 mmHg; P<0.001), and DBP also significantly decreased (DBP: Mean ± SEM: DAPA vs. Ctrl: 0 h: 97.3167 ± 1.89561 mmHg vs. 98.6917 ± 1.89500 mmHg; 2 h: 87.1950 ± 1.83324 mmHg vs. 101.3175 ± 1.28424 mmHg; P<0.01). The SBP and DBP of the DAPA group significantly decreased 2 hours after administration, whereas there were no significant differences between 0 and 2 hours in the control group mice. The HR measurements showed no significant changes between the DAPA and control groups, both before and 2 hours after administration (HR: Mean ± SEM: DAPA vs. Ctrl: 0 h: 417.3413 ± 9.79181 bpm vs. 432.4010 ± 11.75110 bpm; 2 h: 430.9637 ± 8.17309 bpm vs. 415.7540 ± 9.41931 bpm; P>0.05) (**Figure 2D**).

### 5.2 Distribution of SGLT-2 and tracing study of administered DAPA-Cy3

**Figure 3:**
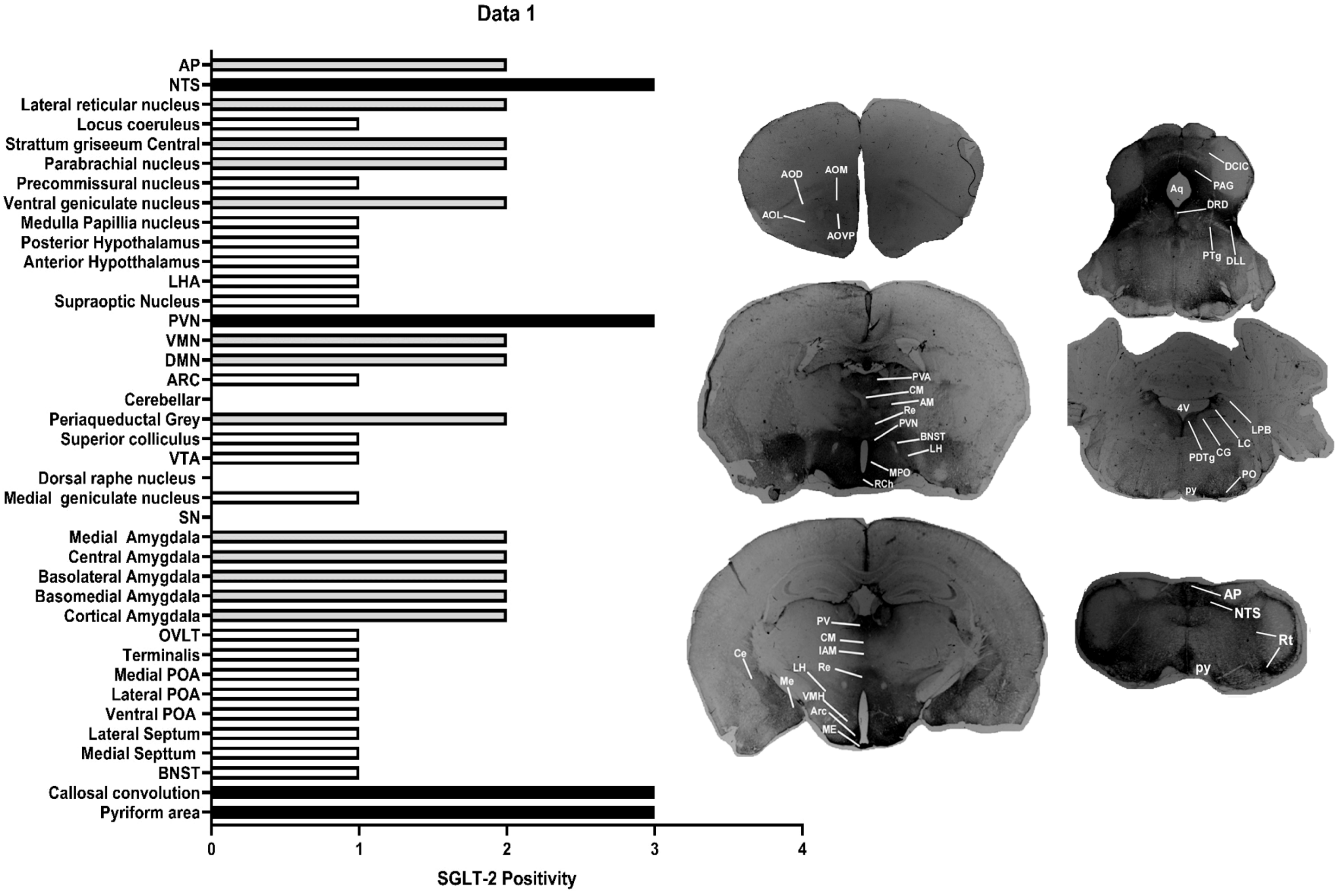
The immunohistochemistry assay showed the distribution of SGLT-2 in key regions of the mouse brain. Significant expression was observed in multiple critical areas of the brain. The immunohistochemical staining was quantified using ImageJ software with a semi-quantitative scoring system (scale: 0-4), where a higher score corresponds to increased density.

**Figure 4:**
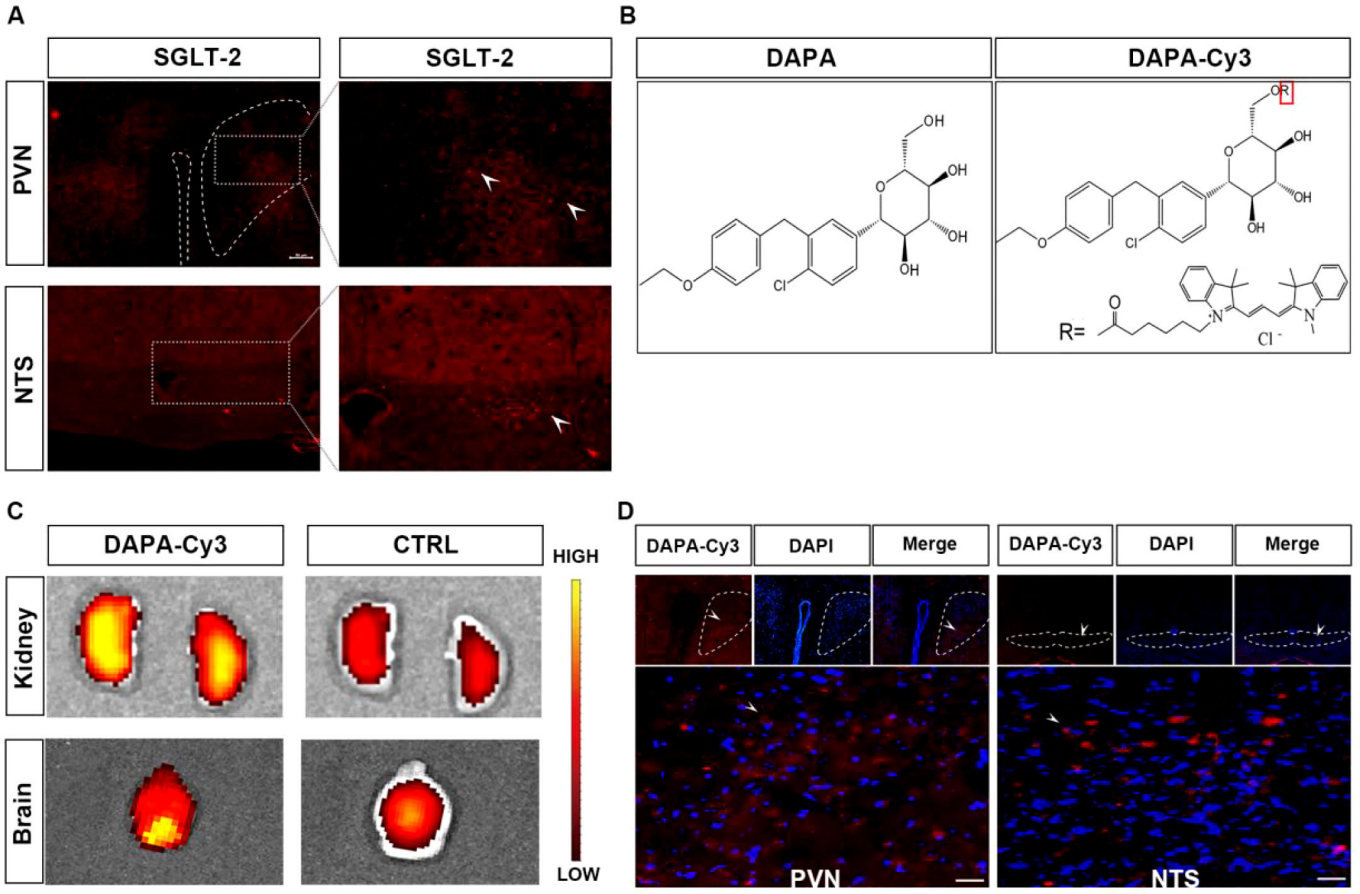
SGLT-2 expression in PVN and NTS, and the imaging reveals the distribution of DAPA-Cy3 in mice. **(A)** The PVN and NTS are regions with high expression of SGLT-2. **(B)** Structure of dapagliflozin and DAPA-Cy3. **(C)** Compared to the control group, the *in vivo* animal imaging results at 2 hours after administering DAPA-Cy3 to mice showed a significant concentration in the kidneys, with distribution also observed in the mouse brain tissue. The bar represent Radiant Efficiency, (pisec/cm^2^/sr) uw/cm^2^ Color Scale Min = 2.00e7MAX = 4.00e7. **(D)** The fluorescence results in mouse brain tissue indicated that DAPA-Cy3 was distributed in the PVN and NTS.

Several studies have confirmed the expression of SGLT-2 in the brain. We mapped the localization of SGLT-2 in key regions of the mouse brain (**Figures 3**, **4A**). Next, we synthesized DAPA-Cy3 with a molecular weight of 883 Da (**Figure 4B**) to test whether it could cross the BBB. *In vivo* imaging showed that DAPA-Cy3 was notably concentrated in the kidneys, with additional distribution seen in mouse brain tissue (**Figure 4C**). Later, the mouse brain tissue was sectioned into 25μm slices and examined under a fluorescence microscope. The results showed that DAPA-Cy3 was present in both the PVN and NTS nuclei, which are strongly linked to BP regulation (**Figure 4D**).

### 5.3 The comparison of the c-Fos expressions in PVN and NTS of mice treated with DAPA and vehicle.

**Figure 5:**
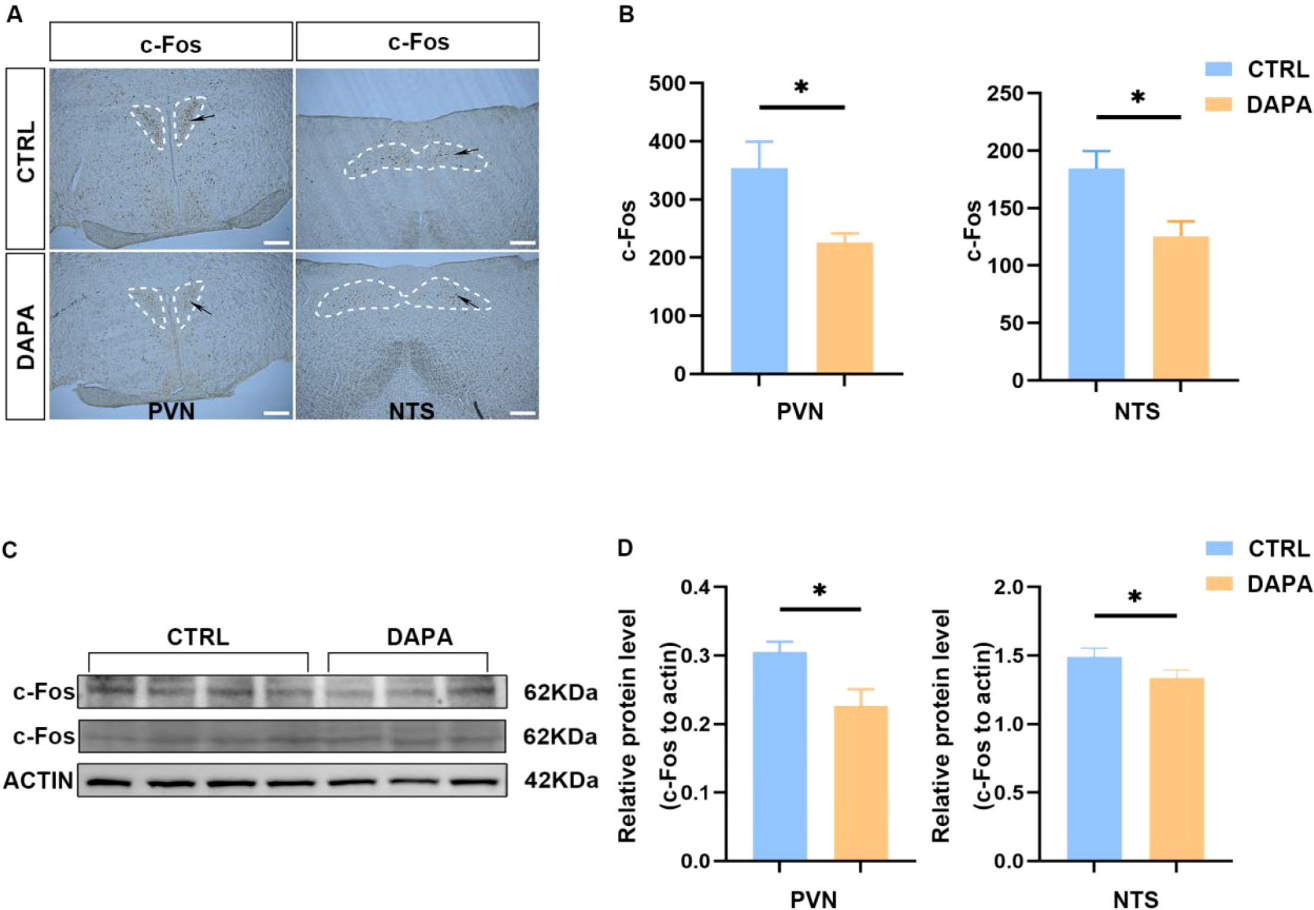
The comparison of expression of c-Fos in PVN and NTS. Immunohistochemistry showed that c-Fos was mildly reduced in the PVN (*P < 0.05, n=3) and NTS (*P < 0.05, n=3) of the DAPA group compared to the control group. **(A, B)** Western blotting results indicated that, relative to the control group, the DAPA group expressed less c-Fos protein in both the PVN (*P < 0.05, n=3) and NTS (*P < 0.05, n=3) **(C, D)**. All data are presented as mean ± SEM.

Subsequently, we examined neural activation in key neural nuclei, including the PVN and NTS. Immunohistochemical results (**Figure 5**) showed that, compared to the control group, c-Fos expression was significantly reduced in the PVN and NTS regions of the experimental group (P < 0.05) (**Figures 5A, B**). Similarly, western blotting (**Figure 5C**) demonstrated a decrease in c-Fos protein levels (P < 0.05) (**Figure 5D**). These findings suggest that DAPA inhibited c-Fos expression in pre-sympathetic neuron populations of the PVN and NTS.

### 5.4 DAPA suppressed the activation of pre-sympathetic neurons in the PVN.

**Figure 6:**
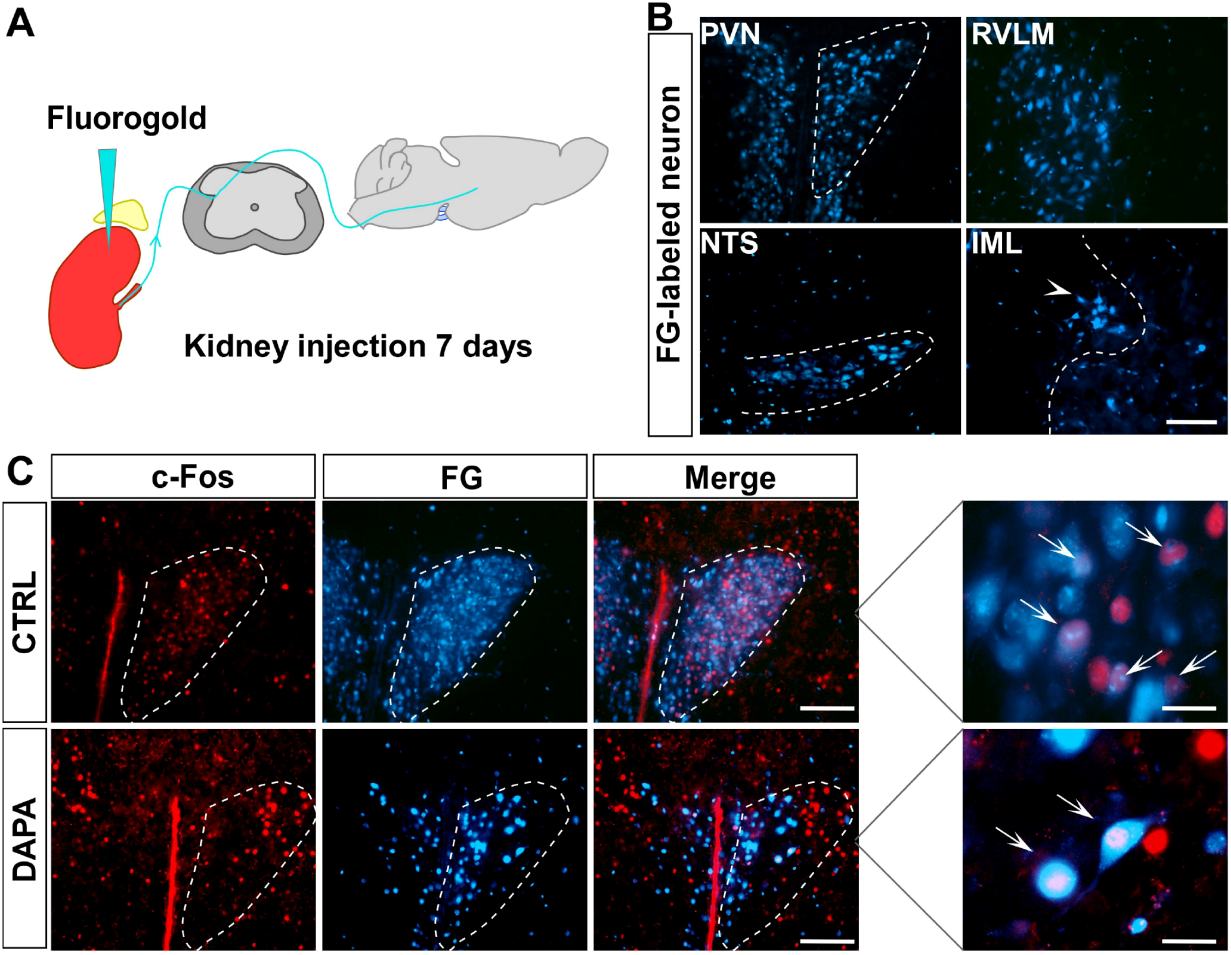
Co-localization analyses show overlapping of retrograde labeling of pre-sympathetic neurons by FG and c-Fos positive neurons in PVN. FG microinjection into the mouse kidney to retrogradely trace the pathway and label the pre-sympathetic neurons in the PVN after 14 days **(A)**; FG retrogradely labeled neurons in the IML, NTS, RVLM, and PVN **(B)**; FG-labeled pre-sympathetic neurons co-localized with c-Fos-positive neurons **(C)**.

Retrograde labeling of neurons associated with the sympathetic nerves was carried out using 4% FG microinjection into the kidneys (**Figure 6A**). Under a fluorescence microscope, neurons in the IML, RVLM, NTS, and PVN were labeled (**Figure 6B**). Immunofluorescence results showed the co-localization of pre-sympathetic neurons and c-Fos-positive neurons in the PVN, where c-Fos was noticeably reduced in the DAPA group (a decrease in c-Fos positivity (8.1% vs. 27%) in the PVN of the DAPA group) (**Figure 6C**).

### 5.5 DAPA increased the expression of nNOS in neurons within PVN.

**Figure 7:**
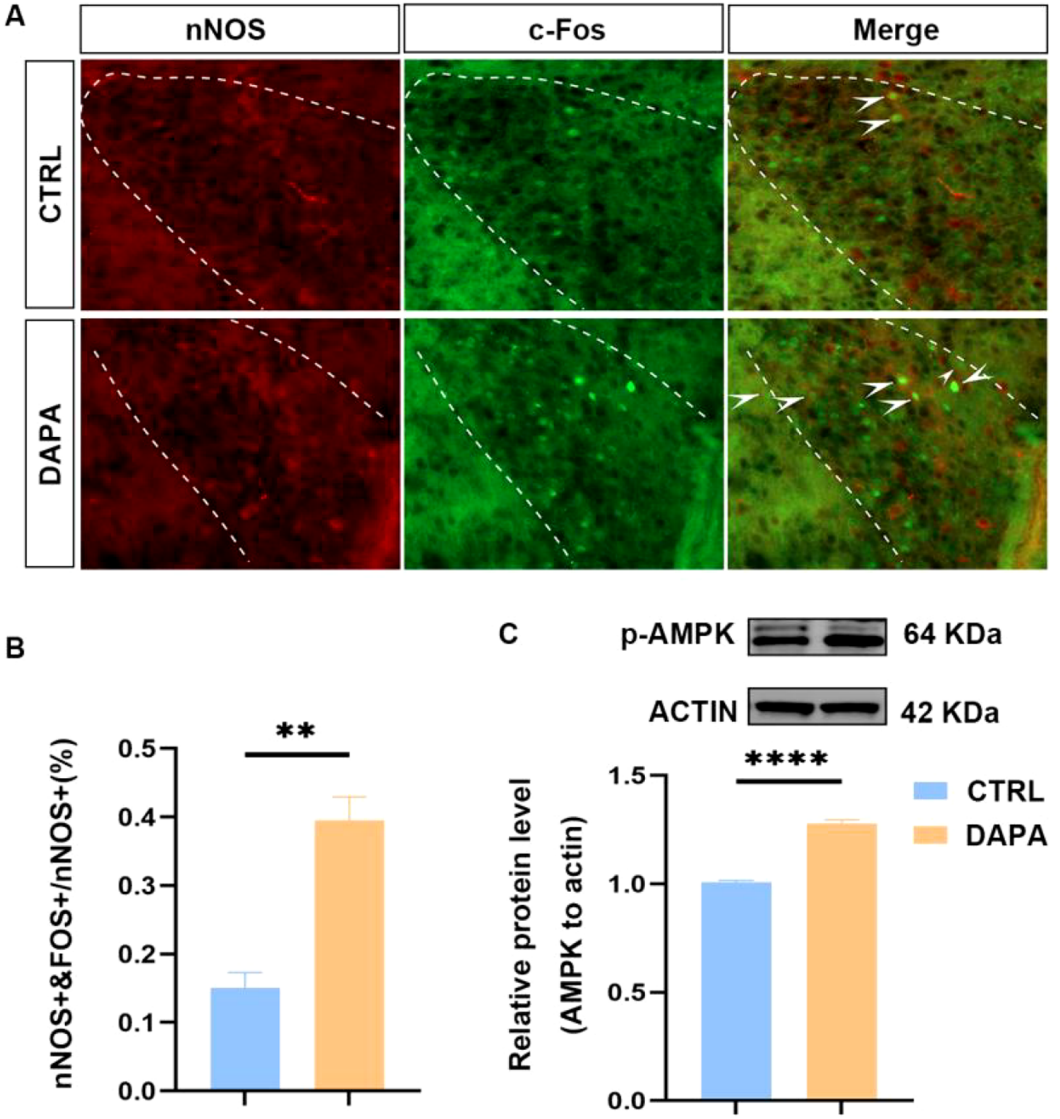
The comparison of DAPA and control group on the increased nNOS expression and co-localization with c-Fos expression in neurons within PVN. The immunofluorescence assay displays and compares the co-localization of PVNnNOS+ neurons and PVN c-Fos+ neurons between the DAPA and control groups **(A)**, demonstrating that the PVNnNOS+/c-Fos+/PVNnNOS+ ratio in the DAPA group was significantly increased (**P < 0.01, n=3) **(B)**, and that the level of p-AMPK protein expression was significantly higher in the DAPA group compared to the control group (****P < 0.0001, n=6) **(C)**.

Using a specific immunofluorescence assay to stain the nNOS neurons in the PVN, their positivity was examined under a fluorescence microscope (**Figure 7**). Using immunofluorescence co-localization techniques, we quantified the number of co-localized PVN-nNOS-positive neurons (PVNnNOS+) with c-Fos-positive neurons (PVNc-Fos+). The results showed that in the DAPA group, the ratio of PVNnNOS+/c-Fos+ to total PVNnNOS+ was higher than in the control group (P < 0.05) (**Figures 7A, B**). Compared to CTRL, DAPA was able to elevate the activation levels of nNOS. A regrettable limitation is our lack of further investigation into the specific mechanism (NO release levels in the brain). Additionally, the results showed an increase in p-AMPK expression via WB assay in the DAPA group (P < 0.05) (**Figure 7C**).

### 5.6 DAPA suppressed the expression of CRH within PVN.

**Figure 8:**
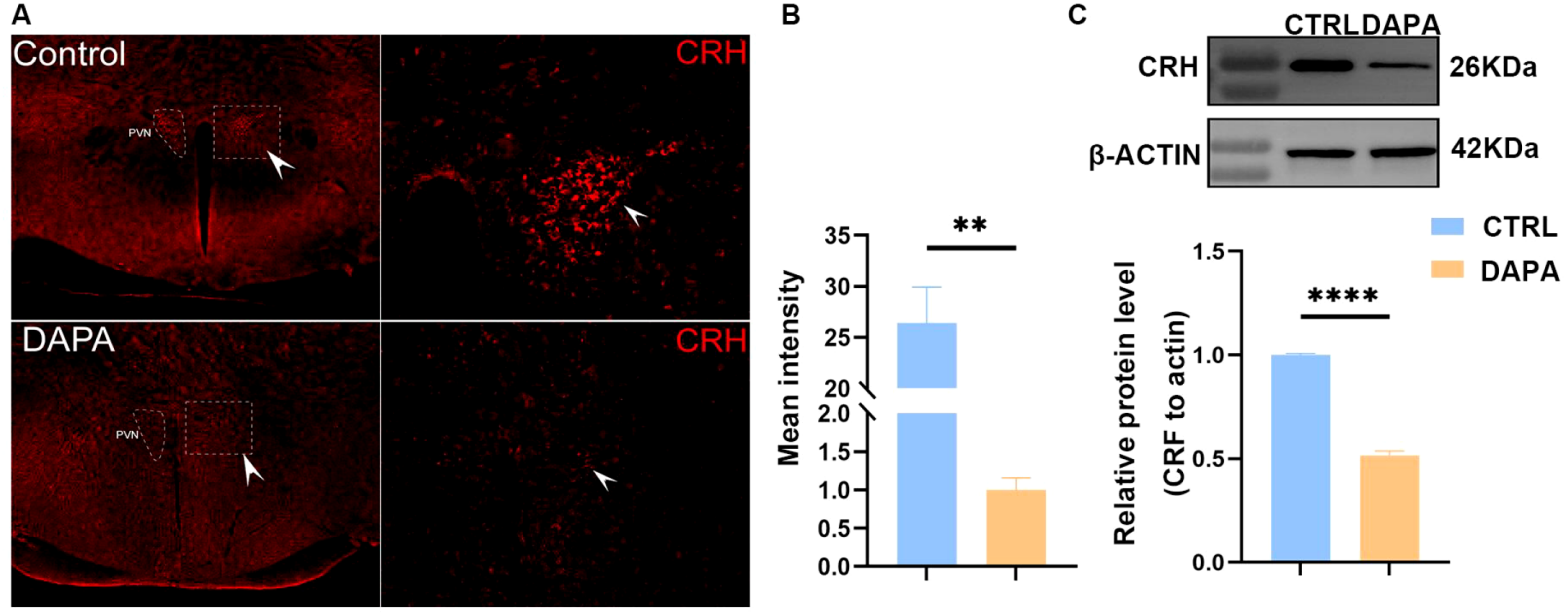
The comparison of CRH expression within PVN between the DAPA and control groups. Immunofluorescence revealed a relatively lower expression of CRH in the PVN brain region of the DAPA and control groups **(A)**. Quantitative analysis showed that the average fluorescence intensity of CRH in the PVN brain region of the DAPA group decreased significantly (**P < 0.01, n=3) **(B)**. The expression of CRH protein in the PVN of DAPA was reduced considerably (****P < 0.0001, n=6) **(C)**.

Using immunofluorescence techniques, CRH-positive neurons (PVNCRH+) in mouse brain tissue were detected (**Figure 8A**). Fluorescence intensity analysis of the positive regions revealed that, compared to the control group, the average fluorescence intensity of CRH in the PVN of the DAPA group was decreased (P < 0.05) (**Figure 8B**). Western blot results (**Figure 8C**) showed a significant reduction in the protein expression level of CRH in the hypothalamus of mice in the DAPA group, indicating that DAPA could decrease CRH synthesis.

### 5.7 DAPA suppressed the expression of VP in neurons of PVN

**Figure 9:**
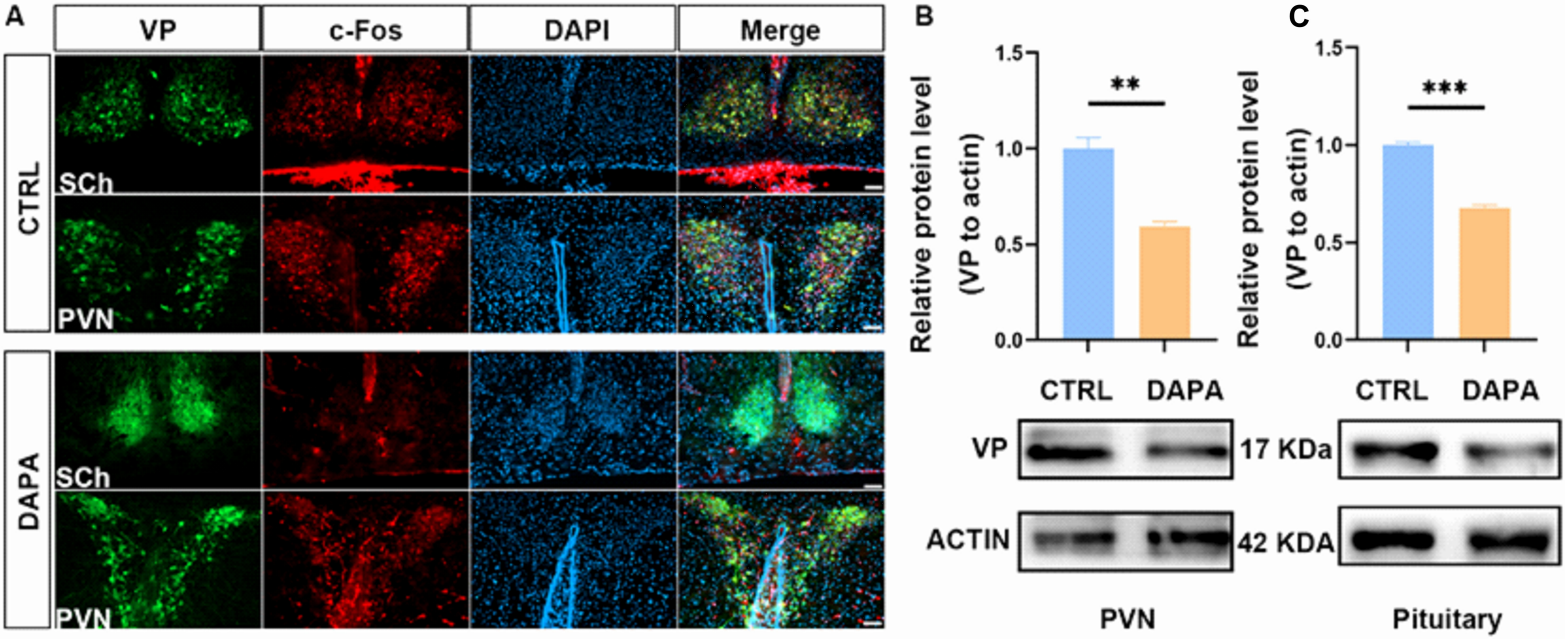
Comparison of VP expression in the hypothalamus between the DAPA and control groups. The co-localization analyses showed reduced PVNVP+/c-Fos and SCh VP+/c-Fos in the DAPA group compared to the control group **(A)**; Western blot analysis revealed that VP protein expression levels in the hypothalamus were reduced in the DAPA group (****P < 0.0001, n=6) **(B)**; VP expression levels in the pituitary gland were also significantly decreased (****P < 0.0001, n=6) **(C)**.

The expression of c-Fos in VP-positive neurons (PVNVP+/c-Fos) was identified using immunofluorescence co-localization techniques. Compared to the control group, the number of PVNVP+/c-Fos and SChVP+/c-Fos cells in the DAPA group was significantly lower than in the control group (**Figure 9A**). Western blot analysis of hypothalamic proteins showed that VP protein levels in the DAPA group were significantly reduced compared to the control group (P < 0.0001) (**Figure 9B**). Similarly, Western blotting of pituitary proteins indicated that VP expression in the DAPA group was markedly lower than in the control group (P < 0.0001) (**Figure 9C**). This decrease could lead to reduced VP release into circulation, causing vasodilation and ultimately lowering blood pressure.

## 6 Discussion

T2DM, a multifactorial metabolic disorder, is a primary risk factor for morbidity and mortality associated with HF, chronic kidney disease (CKD), retinopathy, and peripheral neuropathy (27). Recent studies have shown that the novel antidiabetic drug SGLT-2i offers significant cardioprotective effects, including lowering blood pressure (BP), alleviating heart failure (HF), and reducing the occurrence of adverse cardiovascular events (28, 29). In recent years, the use of SGLT-2i in treating HF among diabetic patients has become a focus of research. However, the specific mechanisms beyond glycemic control and renal physiology are still unclear and need further study. Therefore, we explored the central mechanisms of the sympathetic nervous system (SNS) related to BP and HR regulation influenced by SGLT-2i. Our results indicate that DAPA can access regions such as the hypothalamus, midbrain, and brainstem by crossing the blood-brain barrier (BBB) and interact with specific neurons, including nNOS, CRH, and VP, in the paraventricular nucleus (PVN) that are upstream of SNS regulation, which is closely involved in BP regulation.

In this study, we examined DAPA’s effects on BP and HR. The specific 2-hour post-administration time point for observation was determined based on our pilot data, which indicated that this window coincides with the peak of the most significant physiological effects (e.g., reduction in heart rate, suppression of sympathetic nerve activity) and molecular markers (e.g., c-Fos activation within specific nuclei). Our findings showed that, compared to the control group, mice given DAPA had significantly lower SBP (**Figure 2B**) and DBP (**Figure 2C**), aligning with prior clinical and preclinical studies (30). Additionally, weight loss has been suggested as a possible factor in BP reduction by SGLT-2i (31). However, in our study, the observed BP decrease in mice was more likely due to sympathoinhibition, as no significant weight loss occurred, implying that DAPA may regulate BP through both short- and long-term mechanisms (32). Interestingly, clinical studies in T2DM patients have demonstrated that SGLT-2i-induced BP lowering does not significantly increase HR (33), further indicating that DAPA’s BP-lowering effects are not solely due to its hypoglycemic or osmotic diuretic actions but also involve ongoing neuroendocrinological regulation (34). Consistent with this, our current study found no significant HR changes within 2 hours after administering dapagliflozin compared to the control group (**Figure 2D**). Previous clinical studies comparing diuretics and SGLT-2i in managing HT have shown that SGLT-2i reduces interstitial fluid volume more effectively than diuretics, suggesting that osmotic diuretics play a limited role in HT regulation (35). Similarly, we did not observe significant differences in urine output between the two groups. Some researchers argue that the BP-lowering effect of SGLT-2i isn’t due to osmotic diuresis because the hypotensive response occurs before significant fluid loss (36). Furthermore, Briasoulis et al. demonstrated that SGLT-2i could lower both systolic and diastolic BP, likely through urinary sodium excretion and SNS inhibition (37). The lack of HR changes accompanying BP reduction supports the idea that SNS inhibition contributes to SGLT-2i’s effects, as numerous studies have shown that, unlike diuretics, SGLT-2i do not trigger compensatory HR increases in response to BP drops (38). In obese rats fed a high-salt diet that developed HT, abnormal SNS activity disrupted circadian BP patterns, but SGLT-2i treatment restored both BP and SNS activity rhythms, significantly lowering BP without affecting HR (39), consistent with our findings where BP regulation did not increase HR, confirmed by immunohistochemistry results in the SGLT-2 localization study (**Figure 3**). Additionally, we examined DAPA’s effect on blood glucose in mice and found no impact on normoglycemic animals. We believe the glucose-dependent hypoglycemic effect observed in humans taking DAPA as monotherapy is supported by our findings in normal mice in this study.

Studies have confirmed that SGLT-2 is mainly expressed in the epithelial cells of the S1 and S2 segments of the proximal tubule in the kidney and is responsible for reabsorbing about 90% of glucose from primary urine (40). Our previous studies used a histochemistry assay revealing extensive SGLT-2 expression in multiple areas in normal male C57BL/6 mice. In this experimental study, we also found that SGLT-2 is especially strongly expressed in PVN and NTS (21). Hamed et al. suggested that SGLT-2i, a class of partially lipophilic drugs, can penetrate the BBB (41). To determine the penetration ability of DAPA and whether its distribution matches SGLT-2 expression, we used synthesized DAPA-Cy3 to address this, and observed that, compared to the saline group, DAPA-Cy3 was highly concentrated in the kidneys and brain tissues of mice, particularly in the brainstem and hypothalamus. These areas are largely consistent with the distribution of SGLT-2 expression identified in previous SGLT-2 localization studies (40, 42). Ghezzi et al. employed a novel, noninvasive imaging method with high temporal and spatial resolution to track the binding of radioisotope F-labeled dapagliflozin (F-DAPA) to SGLT-2 proteins on the outer surface of organ cells. The binding of F-DAPA to SGLT-2 in the kidney and brain tissues supports the idea that SGLT-2i can bypass the BBB (43). Pawlos et al. indicated that SGLT-2i can achieve a brain/serum ratio of 0.5 (44), which indirectly supports our findings regarding the distribution of DAPA-Cy3 in the PVN, NTS, arcuate nucleus (ARC), and cerebral cortex.

To further investigate the central actions and pathways of SGLT-2i in regulating the SNS, we examined the effect of DAPA on c-Fos, a marker of neural activation, in different regions of the mouse brain. These results suggest that DAPA may inhibit c-Fos expression in pre-sympathetic neuron populations of the PVN and NTS. Next, we explored the neuronal subtypes upstream of DAPA-SNS regulation within the PVN and relevant neural pathways. Previous studies have shown an imbalance between inhibitory and excitatory synaptic inputs to the PVN in regulating pre-sympathetic neuronal activity, contributing to the pathophysiology of HT, which leads to excessive stimulation of pre-sympathetic neurons in the PVN and increased SNS outflow (45). We used retrograde tracing to label neurons in the PVN and identified the specific pre-sympathetic neurons and the responsible SNS pathway by applying FG to the mouse kidneys, followed by routine DAPA administration 14 days after injection to the labeled mice. As expected, we observed significant overlap between FG-labeled pre-sympathetic neurons and c-Fos+ neurons in the brain, with reduced activation of pre-sympathetic neurons in the DAPA group—as shown by lower c-Fos expression—indicating that DAPA can suppress pre-sympathetic neuron activity. We also found a decrease in c-Fos+ neurons in the NTS in the DAPA group, suggesting that DAPA also affects sympathetic outflow. It is well established that the function of PVN pre-sympathetic neurons is finely regulated by inhibitory (GABAergic) and excitatory (glutamatergic) synaptic inputs (46). However, whether DAPA modulates GABAergic or glutamatergic neurons to suppress PVN pre-sympathetic activity remains unstudied and unresolved.

In the hypothalamus, the localization of neuronal nitric oxide synthase (nNOS)-positive neurons is mainly restricted to the PVN and supraoptic nucleus (SO) (47), where we observed changes in nNOS expression related to SNS activity in our immunofluorescence study (**Figure 7**). An earlier study showed that injecting the NO donor sodium nitroprusside into the PVN decreased sympathetic nerve activity and lowered blood pressure in rats (48). According to Sharma et al., a reduction in nNOS neurons in chronic HF rat models causes a decrease in nitric oxide (NO) in the PVN, leading to a dysregulated sympathetic tone (49). Our results suggest that DAPA increases nNOS expression in PVN neurons, which may be linked to the suppression of pre-sympathetic neuronal activity and downstream outflow to the NTS. A decrease in brain tissue glucose compared to circulating levels can elevate the AMP/ATP ratio in neurons, signaling the activation of AMP-activated protein kinase (AMPK). Partially activated AMPK can phosphorylate nNOS enough to increase NO levels and promote cGMP production. Elevated cGMP enhances full phosphorylation of AMPK through positive feedback (50, 51). Therefore, we propose that DAPA’s local glucose deprivation effect in the hypothalamus, especially in the PVN, may cause partial activation of brain AMPK. Therefore, direct measurement of brain interstitial glucose via techniques such as microdialysis is the key next step to verify this hypothesis. This activation could stimulate certain nNOS neurons to release NO, acting on PVN pre-sympathetic neurons, thereby inhibiting sympathetic outflow and lowering blood pressure in mice. However, technical limitations prevented direct measurement of NO release dynamics in the brain. Future work will directly test the necessity of nNOS in this pathway. Using pharmacological inhibition or genetic knockout of nNOS, we will examine whether key effects of DAPA, such as reduced c-Fos activation or shifts in neuronal excitability, are significantly suppressed.

Studies have shown that overactivation of the HPA axis and SNS plays a role in the development of HT and HF in humans and animal models. CRH-secreting neurons are widely distributed in the PVN, and a positive feedback loop exists between CRH neurons and catecholaminergic autonomic centers in the brainstem, which can influence autonomic outflow (52). We found that mice treated with DAPA experienced a reduction in CRH synthesis and secretion in the hypothalamus. We speculate that this is likely related to the blockade of the CRH-catecholaminergic positive feedback loop, which may lead to decreased autonomic outflow and, consequently, a relatively increased parasympathetic outflow, contributing to the BP-lowering effect. Additionally, CRH neurons in the PVN project to specific neurons in the NTS via the release of CRH and glutamate from axonal terminals, which activates these neurons by binding to CRH receptor 2 (CRHr-2) and glutamate receptors (GluR), respectively, resulting in increased BP (53). This mechanism may be the target of DAPA’s inhibition of CRH expression.

In this study, we measured VP expression levels in the hypothalamus and pituitary gland and found that mice treated with DAPA showed a significant decrease in VP in both areas (**Figure 9**). This decline could lead to reduced VP release into the bloodstream, causing vasodilation and ultimately lowering blood pressure. Additionally, an alternate neural pathway is involved: axons from VP neurons project to the RVLM, NTS in the brainstem, and IML in the thoracic spinal cord, which are key parts of the SNS pathway and important for integrating peripheral sympathetic output (54). Multiple preclinical studies have shown that administering VP to the PVN of normovolemic and normotensive rats significantly raises blood pressure and increases sympathetic activity (15, 55). Our co-localization analysis revealed that VP-positive neurons could be a neural target inhibited by DAPA in the CNS, potentially linked to suppressed SNS outflow via this pathway, as a primary reason for the blood pressure reduction. Furthermore, SChVP can release VP from their dendrites and nerve terminals, influencing diurnal VP rhythms in cerebrospinal fluid (56). Although our study found that DAPA inhibits c-Fos activation in VP neurons of the SCh, which may relate to its blood pressure effects, we did not investigate the diurnal rhythm of VP in cerebrospinal fluid.

## 7 Conclusion

**Figure 10:**
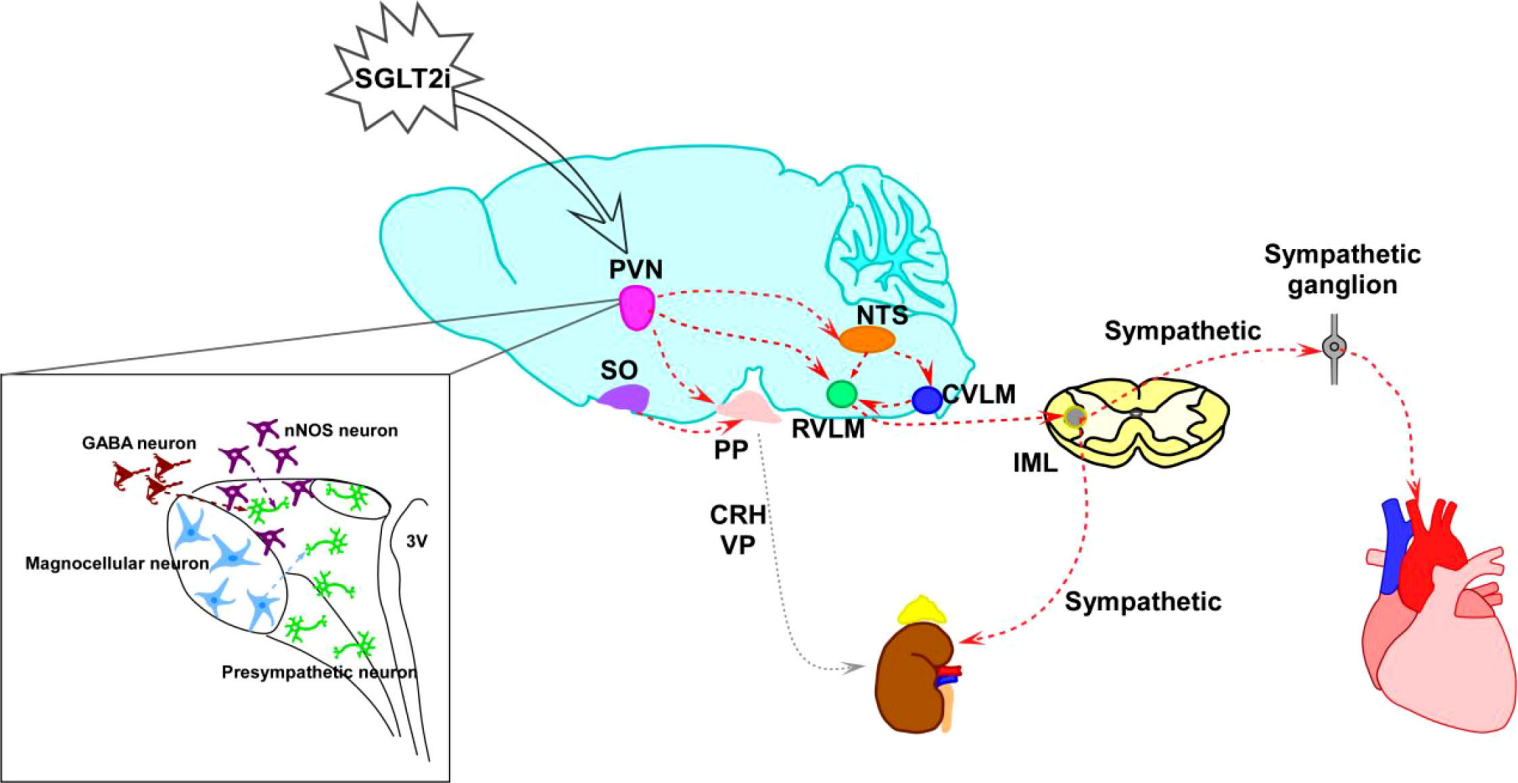
The proposed mechanism of BP and cardiovascular regulation by SGLT-2i. The central nuclear pathways involved in SNS include PVN→NTS→RVLM→IML, PVN→RVLM→IML, and PVN→IML. SGLT-2i may help regulate the nuclei involved in blood pressure and cardiovascular function, such as the PVN, NTS, and RVLM, ultimately lowering blood pressure by inhibiting SNS outflow. Additionally, SGLT-2i can target pre-sympathetic, CRH, nNOS, and VP neurons, suppressing SNS control of cardiovascular activity and reducing blood pressure.

In summary, our mechanistic profiling study demonstrated that DAPA can quickly lower blood pressure in normal male mice without causing significant changes in heart rate. This suggests that the reduction of sympathetic activation may contribute to DAPA’s cardiovascular effects. The study also showed that SGLT-2 is widely expressed in the mouse central nervous system, and DAPA can cross the blood-brain barrier to reach regions such as the hypothalamus, midbrain, and brainstem. Experiments examining the effects of DAPA on brain nuclei and key neuronal subtypes upstream of the sympathetic nervous system may reveal how DAPA influences blood pressure regulation. DAPA appears to regulate cardiovascular activity through neuroendocrine mechanisms, likely involving interactions between DAPA and pre-sympathetic, CRH, nNOS, and VP neurons (**Figure 10**). It acts on CRH neurons, inhibiting their synthesis and release, which suppresses sympathetic activity and the HPA axis to lower blood pressure. Additionally, DAPA can act on nNOS neurons that release NO to inhibit sympathetic control of the cardiovascular system, further reducing blood pressure. Exploring the connection between the CNS and SGLT2 inhibitors could lead to new and effective treatments for cardiovascular diseases. Further research is needed to identify the specific neural circuits and molecular mechanisms involved.

## 8 Limitation

It must be emphasized that this study was conducted in normal glucose mice models, with the main purpose of isolating and identifying the direct central effects of DAPA without interference from hyperglycemia and severe metabolic disorders. Therefore, our findings should be regarded as a proof of concept under normal physiological conditions. The unique neuroendocrine and sympathetic nervous environments present in diabetes or heart failure patients may alter the response to DAPA. Future studies in disease models are crucial for confirming the clinical relevance of these findings. Another key limitation of this study is the exclusive use of male mice. Future investigations are warranted to determine whether the central sympathetic and neuroendocrine effects of DAPA are conserved or divergent in females, which will be critical for understanding its therapeutic application across both sexes.

## Data Availability

The raw data supporting the conclusions of this article will be made available by the authors, without undue reservation.
